# Role of norms in variation in cancer centers’ end-of-life quality: qualitative case study protocol

**DOI:** 10.1186/s12904-020-00641-x

**Published:** 2020-08-27

**Authors:** Kristin E. Knutzen, Karen E. Schifferdecker, Genevra F. Murray, Shama S. Alam, Gabriel A. Brooks, Nirav S. Kapadia, Rebecca Butcher, Amber E. Barnato

**Affiliations:** 1 Department of Behavioral, Social, and Health Education Sciences, Rollins School of Public Health, Emory University, Atlanta, GA USA; 2 The Dartmouth Institute for Health Policy and Clinical Practice, Geisel School of Medicine, Dartmouth College, Lebanon, NH USA; 3 Department of General Internal Medicine, Boston Medical Center, Boston, MA USA; 4 Evidera, Pharmaceutical Product Development, Bethesda, MD USA; 5 Department of Medicine, Geisel School of Medicine, Hanover, NH USA; 6 Norris Cotton Cancer Center at Dartmouth-Hitchcock Medical Center, Lebanon, NH USA

**Keywords:** End-of-life, Norms, Heuristics, Cancer, Minority health

## Abstract

**Background:**

A critical barrier to improving the quality of end-of-life (EOL) cancer care is our lack of understanding of the mechanisms underlying variation in EOL treatment intensity. This study aims to fill this gap by identifying 1) organizational and provider practice norms at major US cancer centers, and 2) how these norms influence provider decision making heuristics and patient expectations for EOL care, particularly for minority patients with advanced cancer.

**Methods:**

This is a multi-center, qualitative case study at six National Comprehensive Cancer Network (NCCN) and National Cancer Institute (NCI) Comprehensive Cancer Centers. We will theoretically sample centers based upon National Quality Forum (NQF) endorsed EOL quality metrics and demographics to ensure heterogeneity in EOL intensity and region. A multidisciplinary team of clinician and non-clinician researchers will conduct direct observations, semi-structured interviews, and artifact collection. Participants will include: 1) cancer center and clinical service line administrators; 2) providers from medical, surgical, and radiation oncology; palliative or supportive care; intensive care; hospital medicine; and emergency medicine who see patients with cancer and have high clinical practice volume or high local influence (provider interviews and observations); and 3) adult patients with metastatic solid tumors and whom the provider would not be surprised if they died in the next 12 months and their caregivers (patient and caregiver interviews). Leadership interviews will probe about EOL institutional norms and organization. We will observe inpatient and outpatient care for two weeks. Provider interviews will use vignettes to probe explicit and implicit motivations for treatment choices. Semi-structured interviews with patients near EOL, or their family members and caregivers will explore past, current, and future decisions related to their cancer care. We will import transcribed field notes and interviews into Dedoose software for qualitative data management and analysis, and we will develop and apply a deductive and inductive codebook to the data.

**Discussion:**

This study aims to improve our understanding of organizational and provider practice norms pertinent to EOL care in U.S. cancer centers. This research will ultimately be used to inform a provider-oriented intervention to improve EOL care for racial and ethnic minority patients with advanced cancer.

**Trial registration:**

Clinicaltrials.gov;  NCT03780816; December 19, 2018.

## Background

The National Academy of Medicine has identified increasingly aggressive, burdensome and expensive end-of-life (EOL) treatment as a major public health problem [1]. The American Society of Clinical Oncology and National Quality Forum (NQF) define aggressive, burdensome, and expensive EOL treatment in cancer as the receipt of chemotherapy in the last 14 days of life (NQF #0210), intensive care unit (ICU) admission in the last 30 days of life (NQF #0213), and non (NQF #0215) or late (NQF #0216) hospice referral [2]. Such treatment adversely affects patient quality of life, quality of dying, and caregiver bereavement outcomes [3–6]. Minorities are more likely to receive such EOL treatment, [7–9] potentially in disproportion to their preferences [10, 11].

Despite attention focused upon integrating early palliative care into advanced cancer treatment, [12–15] ICU admission in the last 30 days of life and late hospice referral have been secularly increasing [16]. Yet not all organizations are equal: cancer centers vary by more than two-fold in these EOL intensity measures [17, 18]. These variations cannot be explained by structural characteristics or case-mix [18]. Since centers serving a higher proportion of minority patients have systematically higher EOL intensity than centers serving a higher proportion of white patients, [11] these variations in practice patterns may contribute to racial disparities in burdensome treatment near death. Moreover, despite efforts to attribute such variation to differences in patient preferences rather than racial disparities, [19] region-level analyses suggest that the impact of these preferences on variation are likely very small [10, 20].

We posit that a critical barrier to improving the quality of EOL cancer care in the US – and among minorities in particular – is our lack of understanding regarding the mechanisms underlying cancer center variation in EOL treatment intensity [21]. The overarching hypothesis driving this study is that differences in local organizational and provider social norms – rules about which there is at least some degree of consensus, enforced through social sanctions [22] – are a key mechanism underlying this variation. We base this hypothesis on our preliminary work at two US academic medical center hospitals at opposite extremes of EOL treatment intensity demonstrating marked differences in norms of ICU and life-sustaining treatment decision making. These norms were found to directly [23] and indirectly [24] (via influencing patient and family treatment expectations and provider decision making heuristics) affect treatment decisions for minority and non-minority patients with advanced cancer. Norms are fruitful for study because, once understood, they are potentially malleable through explicit leadership efforts and implementation of new forms of positive and negative sanctions via social marketing interventions [25].

Our study aims to study mechanisms underlying cancer center variation in EOL treatment intensity among minority and non-minority patients using a qualitative, case study design and has two objectives. First, we will identify the local organizational and provider practice norms that influence decisions about later-line chemotherapy, hospice, and ICU use among minorities with advanced cancer at major US cancer centers. Second, we will assess the influence of these norms on patient and family expectations and provider decision making heuristics for later-line chemotherapy, hospice, and ICU use among minorities with advanced cancer at major US cancer centers. Below we describe our qualitative study design approach to meet our study objectives.

## Methods/design

### The design of the study

We chose a qualitative case study design at six sites to identify local organizational and provider practice norms that influence variation in EOL treatment intensity, particularly for minority patients. Based on 2016 Medicare claims data analyses, [26] we will recruit 6 of the 11 NCI/NCCN designated cancer centers serving at least 15% African American advanced cancer patients. We based our sample size on recent literature related to sample size sufficiency, recommendations to reach multi-site data saturation, and qualitative research expertise of our study team. First, our study is guided by a theory based on previous research and uses data from multiple sources to test and cross-check for confirming or disconfirming evidence of our theory, a necessary component of ensuring data sufficiency [27, 28]. In addition, conducting qualitative case studies at six sites, each of which will include over 30 interviews plus multiple observations, will produce fine-grained and rich descriptive analysis to generate and compare theoretical insights across sites, as well as across stakeholders (e.g., providers, patients) within sites [29–31]. Our target numbers for interviews and observations are well within recommendations for reaching data adequacy and saturation given our well defined study aim [32]. Lastly, research suggests that qualitative research expertise, including the quality and depth of interview process, is an important criterion to consider for assessing sample size [33]. Our site visit team has over 40 years of collective experience conducting qualitative research in healthcare topics and settings, ensuring a rigorous and thorough process at each of the six sites.

We will target National Comprehensive Cancer Network (NCCN) and National Cancer Institute (NCI) Comprehensive Cancer Centers for our study because they set national standards for high quality cancer care. We will engage up to six sites serving a high proportion of African American patients, ranging in EOL care intensity, and theoretically sampled, to maximize our ability to compare and contrast organizational and provider practice norms related to EOL care. We defined high proportion of minority patients as > 15%. We measured EOL care intensity based upon risk-adjusted metrics of EOL quality using 2016 Medicare fee-for-service claims data: receipt of chemotherapy in the last 14 days of life (NQF #0210), intensive care unit (ICU) admission in the last 30 days of life (NQF #0213), and non (NQF #0215) or late (NQF #0216) hospice referral. Our approach to calculating these EOL quality metrics has been published elsewhere [26]. Given the multivariable nature of these metrics, we use data visualization to purposively select sites for case study that maximize potential heterogeneity in practice patterns [34]. Following the principles of positive deviance sampling, [35, 36] we will sample higher versus lower EOL quality sites in a ratio of 2:1.

We will employ qualitative case study research methods in this study, including inpatient observation procedures and provider, patient, and leadership semi-structured interviews, that have been previously developed and piloted [23, 24, 37–39]. We will augment these methods with outpatient and tumor board observation procedures, which we developed and tested at a non-study NCI-designated cancer center serving a white, rural population. We will iteratively revise all data collection procedures based on researcher experiences and thematic insights following each sampled case study site visit.

### Qualitative data collection

The study team will collect 3 types of data: field notes from direct observation of inpatient and outpatient cancer care and cancer tumor boards; transcribed audio-recorded semi-structured interviews with cancer center leadership, providers, and patients, family members, and caregivers; and artifacts (Table 1). We will link all data from observations using a unique identification (ID) number. We will use a data collection form for field observations to capture provider and patient demographic information, as well as location, time, and individuals present at the encounter. In addition to relevant clinical data, observers will note socio-linguistic dimensions such as turn taking, tone, affect, body positioning, and eye contact. Artifacts collected during the site visit will include workflows, marketing/informational materials, orientation guidelines, quality reporting, and communication documents used in the cancer center.

**Table 1:** Data Collection Rationale

Data Collection Method	Rationale
Direct observation	To learn about EOL care for minority patients with advanced cancer, specifically how it is influenced by:
	1. Organizational and provider practice norms
	2. Provider decision making heuristics
	3. Patient and family expectations
Semi-structured interviews	
Leadership	To probe organization-level:
	1. Norms, including resources, programs, and policies
	2. Site-specific workflows and scheduling logistics
Providers	To explore individual-level:
	1. Motivations, decision heuristics, and/or rationalizations
	2. Unconscious beliefs and assumptions that structure advanced cancer decision making, using case vignettes to prime mental models
Patients, family members, caregivers	To probe individual-level:
	1. Preferences for cancer care
	2. Past, current, and future decisions related to cancer care
Artifact collection	To learn how the organization standardizes workflows, marketing/informational materials, orientation guidelines, quality reporting, and communication documents used in the cancer center, and how this impacts:
	1. Local organizational and provider practice norms
	2. Provider decision making heuristics
	3. Patient and family expectations

Semi-structured interview guides for site leadership focus on institutional norms, including resources, programs, and policies related to EOL care and outcomes, as well as site-specific workflows and scheduling logistics in preparation for site visits. Cognitive mental models semi-structured interview guides for providers, which include EOL case vignettes, explore motivations, decision heuristics, and/or rationalizations [40]. Six EOL vignettes were developed by a medical oncologist, radiation oncologist, and palliative care providers to highlight key decision points common to outpatient or inpatient providers (see Table 2); each vignette has one version with a photo of an African American patient and one with a photo of a white patient. Providers will view one African American case and one white patient case to facilitate mental models debriefing and uncover unconscious beliefs and assumptions related to race that structure advanced cancer decision making. Semi-structured interview guides for patients, family members, and caregivers probe past, current, and future decisions related to their cancer care.

**Table 2:** Vignette Summaries

Vignette Number and Patient Race	Setting/Specialty	Vignette Summary and Key Question
1. African American	Inpatient	**Summary:** 71 year-old man with metastatic gastric cancer. He was living in a skilled nursing facility after a long hospitalization for infection. He is now hospitalized with recurrent fever, respiratory distress, and anxiety.
2. White		**Key Question:** How to manage anxiety and respiratory distress in a patient with advanced cancer and high risk for short-term death.
3. African American	Inpatient	**Summary:** 68 year-old woman with recurrent, metastatic pancreatic cancer and mild dementia. She is scheduled to start palliative chemotherapy next week. She presents to the ED with declining performance status, decreased appetite, and abdominal pain. Her hospital evaluation demonstrates poor kidney function, low blood pressure, and rapid breathing – all worrisome for rapid constitutional decline.
4. White		
		**Key Question:** How to manage a patient with an aggressive cancer presenting to the emergency department with multiple signs of constitutional decline.
5. African American	Medical Oncology	**Summary:** 75 year-old man with advanced, metastatic colon cancer. He is married and lives at home with his wife. He presents to clinic with pain, weight loss, and signs of cancer progression. He asks, “Do you think the chemo is working?”
6. White		**Key Question:** How to answer patient questions about prognosis and next steps in treatment of advanced cancer with limited treatment options.
7. African American	Radiation Oncology	**Summary:** 75 year-old man with a new diagnosis of metastatic renal cell carcinoma. He presents with seizures, brain metastases, and lung metastases. He is unmarried and without children. His performance status is poor and he is not able to make his own health care decisions. His eldest brother is his durable power of attorney, and asks, “Doc, what would you do if he was your brother?”
8. White		
		**Key Question:** How to approach surrogate decision making about management approach for a patient with poor prognosis.
9. African American	Surgical Oncology	**Summary:** 73 year-old man with newly diagnosed non-metastatic lung cancer. He has severe lung disease and significant vascular disease from heavy smoking. He is a poor surgical candidate. He mentions that the stress of his cancer diagnosis has caused him to drink alcohol more heavily than usual and he is coughing up about 1–2 tablespoons of bright red blood daily.
10. White		**Key Question:** How to approach a patient with a new diagnosis of a potentially curable cancer when there are a number of red flags that the patient may do poorly with surgical treatment.
11. African American	Outpatient	**Summary:** 68 year-old woman with a recent diagnosis of pancreas cancer. She has been hospitalized with weight loss, pain, and declining activity. Her evaluation shows a “borderline resectable” pancreatic cancer (initial treatment would be chemotherapy or chemoradiation, if she could tolerate this). She has been unable to eat or ambulate for the last five days, due to poor appetite and performance status. She says, “I’m a fighter, not a quitter” and “With Jesus, anything is possible.” She then asks, “What comes next?”
12. White		
		**Key Question:** How to approach a patient who has a “treatable” diagnosis, but who does not have the performance status to tolerate treatment.

Site visit teams will consist of 2–3 researchers. We will identify a site-specific principal investigator (PI) at each site to help facilitate support from site leadership, and identify and recruit informants for pre site-visit interviews, and providers for site-visit observation and interviews. Up to two months in advance of the site visit, the study team will conduct leadership interviews by phone, including physician and nursing leaders, outpatient oncology practice managers, and key referral service line leaders from palliative/supportive care, hospital medicine, and intensive care. We will approach other site leaders for interviews at the suggestion of the site PI. When necessary, leadership interviews will take place during and after site visits.

**Fig. 1:**
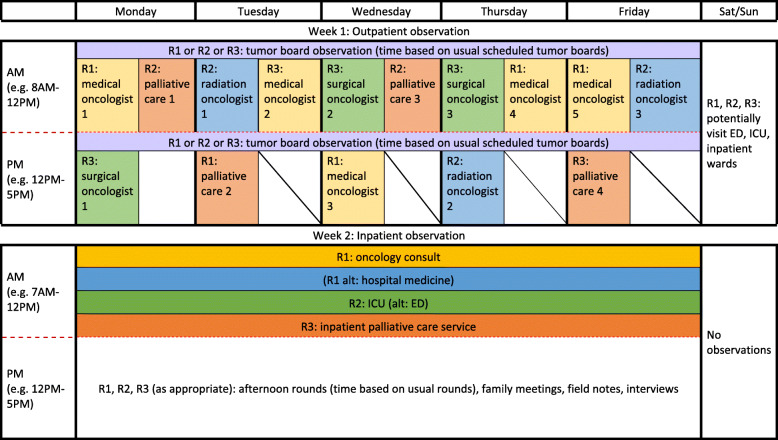
Mock On-Site Observation Schedule. Researchers will ideally observe relevant outpatient clinics during week 1, and inpatient services during week 2. Researchers will go to tumor boards attended by consented providers, as well as other relevant staff meetings (e.g., fellows meetings). Researchers will observe providers during either AM or PM blocks, using the alternating daily block to dictate field notes and conduct interviews with providers and patients on-site

One month in advance of the site visit, the study team will recruit providers for observation in the inpatient and outpatient setting. The observation schedule will involve one researcher assigned to each observed provider for a half-day observation in outpatient clinics (morning or afternoon session), the emergency department (by shift), and inpatient setting (by timing of daily service or consult rounds), as well as scheduled tumor boards and family meetings. Selection criteria for providers to observe and interview focus on maximizing our ability to assess provider norms for advanced cancer care within the particular institutional context that we will explore during leadership interviews. Provider selection criteria includes providers who manage patients with: 1) metastatic solid tumors (i.e., we excluded leukemia, lymphoma, and bone marrow transplant providers) and have either 2) high volumes of patients and/or 3) high peer influence, as perceived by the site PI or other key informants. We seek to recruit medical, radiation, and surgical oncology providers as well as palliative/supportive care providers who see cancer patients (see Table 3 for target sampling frame). We also seek to recruit providers from intensive care, hospital medicine, and emergency medicine who care for acutely ill cancer patients. We present an example observation schedule in Fig. 1. We will ask all providers recruited for observation to complete an interview. Interviews with providers will occur in person during or by phone after the site visit. We will digitally record all interviews and compensate all providers for participating in an interview.

**Table 3:** Target Sampling Frame at Each Site

Data collection setting	N
Outpatient	
Medical oncology	3–5
Surgical oncology	3–5
Radiation oncology	3–5
Supportive/Palliative care	3–5
Emergency medicine	1–3
Inpatient	
Hospital medicine	1–3
Intensive care	1–3
Supportive/Palliative care	1–3
Oncology consult	1–3

At least 2 weeks prior to the site visit, we will send flyers about the study with photos of the study team to the site PI who will facilitate posting of the flier in public settings such as waiting rooms, clinic rooms, infusion suites, and inpatient units. The purpose of this flyer is to alert non-consented individuals to our study purpose and to provide instructions for opting out.

During patient care observation, researchers will directly approach patients and their family/caregivers following introduction by the consented provider. If patients or their family/caregiver verbally consent to be interviewed, the study team member will obtain contact information to arrange for a phone interview at the patient, family member, or caregiver’s convenience at a later date. Selection criteria for patient interviews includes: 1) adults aged 21 years or older; with 2) metastatic solid tumor; 3) whom the provider would not be surprised if they died in the next 12 months; and 4) seen by a consented provider. We will seek to recruit equal numbers of minority and non-minority patients. We will digitally record all interviews and compensate each participant for participating.

### Qualitative data analysis

We will use a qualitative and mixed methods data analysis platform, Dedoose, to manage and analyze all transcribed field notes, interviews, and artifacts, and link relevant data to contextual information (e.g., patient and provider race; site features) (Sociocultural Research Consultants, LLC). We will develop a codebook first deductively, using our theoretical model, findings in the literature, and prior research and then inductively, through an iterative process of close readings and discussion of the data in order to identify additional codes. Three qualitative researchers will apply the codebook to the data, two who will divide and code all the data, and one who will assess reliability of coding by reviewing a subset of the coded data. All three qualitative researchers will discuss differences in coding and resolve by consensus. We will repeat the analysis process after each site visit, to conduct constant comparative analysis regarding similarities and differences between and within sites in support of study aims 1 and 2. After completion of each site visit, the study team will develop a written summary of preliminary quantitative and qualitative findings specific to the site, which will then be sent to all participants from that site, to assess initial validity of the site-specific findings. After completion of all site visits and analysis of data, we will send final study reports to participating sites.

### Rigor and reproducibility

Our research team is also conscious of conducting purposefully informed and respectful research on the cancer experiences of racial and ethnic minorities, and we have taken steps to ensure scientific rigor of our approach and results through study design development and will continue to do so through data collection and data analysis. Based in a relatively non-racially diverse geographic region, our team (*N* = 12) is comprised of 25% racial and ethnic minority researchers. As such, we seek to incorporate greater diversity of racial and ethnic knowledge, as well as disciplinary perspective, through an external advisory board with deep topical expertise in cancer care, palliative care, racial and ethnic health equity, and social norms. Additionally, to address potential researcher bias, the entire study team completed implicit bias training focused on unconscious biases related to attitudes about race, ethnicity, cancer, cancer treatment, death, and dying. One researcher participating in data collection will remain blinded to sites’ EOL treatment intensity classification until data collection is complete.

Finally, we will employ multiple methods of triangulation to assure comprehensiveness and validity of data. Two to three researchers of a multidisciplinary team will participate in each site visit, and an additional three researchers will conduct qualitative analysis, to satisfy investigator triangulation. Method triangulation will include direct observation, semi-structured interviews, and artifact collection. We will achieve data triangulation by observing and interviewing leadership personnel, providers, and patients, family members, and caregivers at each site, of various backgrounds, specialties, and diagnoses, respectively. Qualitative analysis will use both deductive and inductive methods to achieve theory triangulation.

### Ethics approval and consent to participate

The study has been approved by the Dartmouth College Committee for the Protection of Human Subjects (STUDY00031129) and is considered minimal risk. All participating sites will 1) waive independent IRB approval in favor of acknowledging Dartmouth’s IRB, 2) rely on Dartmouth’s IRB via a SMART IRB reliance, or 3) conduct a local ethical review and approval. We will obtain a waiver of informed consent for participant observation; all providers will provide written electronic consent for observation and interview, and all interviewed leadership, patients, and families will provide oral consent for interview. We have obtained a certificate of confidentiality from the National Institutes of Health (NIH) for this study.

We will not record any identifiable or personal information about providers, patients, family members, caregivers, or staff in field notes, except demographic information. A unique ID number will link data from observations and interviews, including demographic data, to consented participants. The key linking the ID number and identifying information of the consented participants will be maintained on a password-protected server. Only the research team will have access to the linkage file. All data collected on individuals will be linked to their ID number alone. We will audio-record and transcribe all handwritten field notes without any identifiable information. We will store all original field notes in a locked filing cabinet, and all transcripts on a password-protected server, to which only the research team will have access. We will give a discreet lapel pin to all providers, staff, patients, family members, and caregivers who do not wish to be observed, as advertised by the informational flyers posted prior to the study team’s arrival at the site. We will not document any individual wearing such a pin in field notes, nor will we approach them for an interview.

We will give all individuals participating in interviews an information sheet prior to the interview, and we will obtain informed consent verbally at the time of the interview. We will obtain informed consent verbally as many of the interviews will be conducted by phone, either before or after the site visit. The process of obtaining verbal consent has been approved by the Dartmouth College Committee for the Protection of Human Subjects. We will record all interviews, and later professionally transcribe them without any identifiable information. We will store all recordings and transcripts on a password-protected server, to which only the research team will have access. Additionally, we consulted the guidelines for end-of-life research put forth in the “Methods of Researching End of Life Care” (MOREcare) project while designing the protocol for this study [41]. Specifically, we considered the risks (e.g., interviewee distress) and rewards (e.g., potential therapeutic effect) that qualitative interviews may have for patients, family members and care givers while designing these protocols.

## Discussion

Our study is the first comprehensive, qualitative study of local organizational and provider norms at minority-serving NCCN and NCI-designated comprehensive US cancer centers. If the aims of this study are achieved, we expect to identify targets for institutional change at cancer centers with lower EOL quality metric performance. The two main deliverables of this research will be 1) knowledge regarding norms and their impact on EOL decision making at participating cancer centers, and 2) identification of potential members of a community research advisory board to oversee future institution-level interventions aimed at improving EOL care. Respective to the first deliverable, participating cancer centers will receive a customized report of our findings about their own center following completion of our site visit. After completion of all site visits, we will work with the American Cancer Society and participating cancer centers to identify local chapters of the American Cancer Society (ACS) and provider medical societies (e.g., county medical and nursing societies) at which we can discuss our findings and their implications for local patients and providers.

Respective to the second deliverable, we anticipate the opportunity to develop institution-level interventions aimed at EOL care in the future. Norms are fruitful for study because, once understood, they are potentially malleable through explicit leadership efforts and implementation of new forms of positive and negative sanctions. Specifically, social marketing – the use of marketing principles to influence human behavior to improve health or benefit society [25] – is a promising strategy for changing norms. Interventionists have successfully applied the principles of social marketing to change HIV risk behaviors [42, 43] and palliative care consultation use [44]. Integrating social marketing interventions in cancer centers with high intensity EOL care could have the effect of improving the quality and cost of cancer care, particularly for racial and ethnic minorities. Further, by studying norms of decision making among groups of physicians, this project will overcome the limitation of past research, which uniformly has neglected this important issue.

## Data Availability

End-of-life metrics data can be found at https://www.dartmouthatlas.org/interactive-apps/end-of-life-cancer-care/. Qualitative data will be de-identified and made available to researchers through the NINR-funded Palliative Care Research Cooperative qualitative data repository after analyses in support of the primary aims are complete. All materials and instruments developed for this study are available by request of the authors.

## Abbreviations

NCCN: National Comprehensive Cancer Network
NCI: National Cancer Institute
EOL: end-of-life
NQF: National Quality Forum
ICU: Intensive care unit
ID: Identification
PI: Principal investigator
IRB: Institutional Review Board
ACS: American Cancer Society
